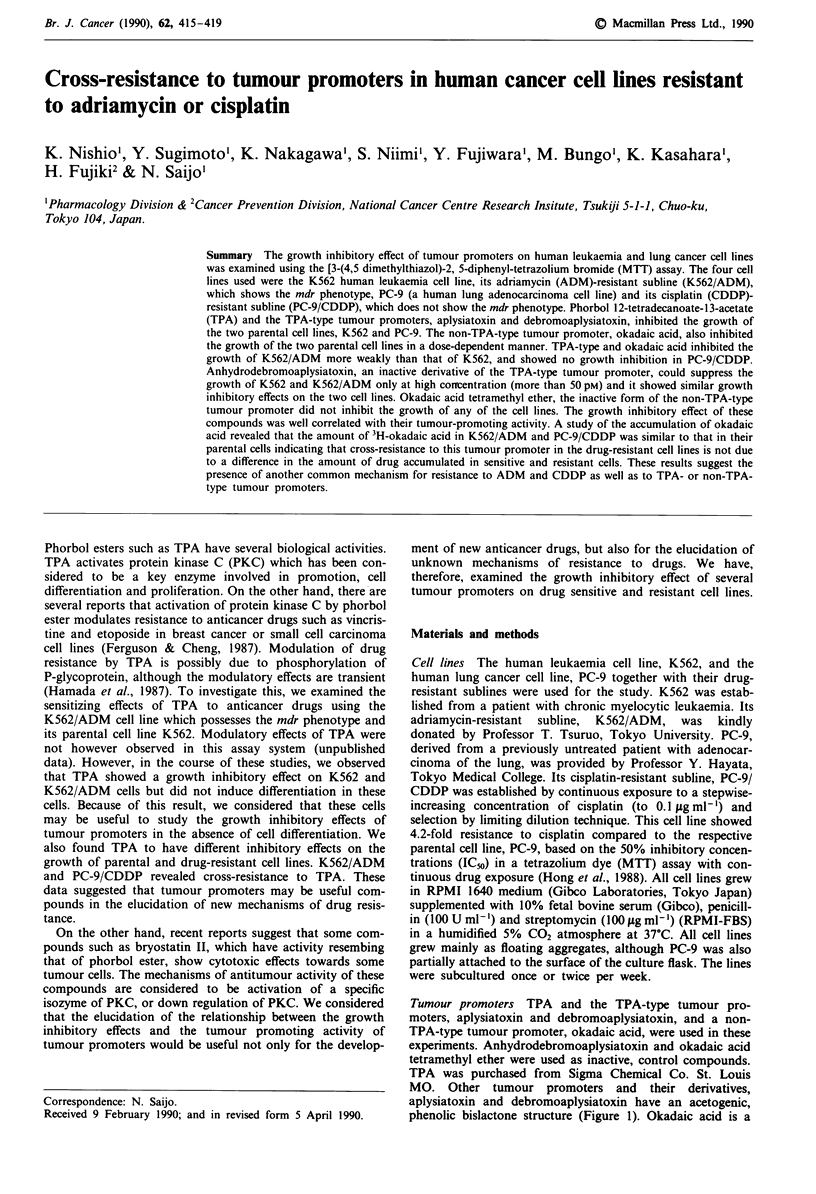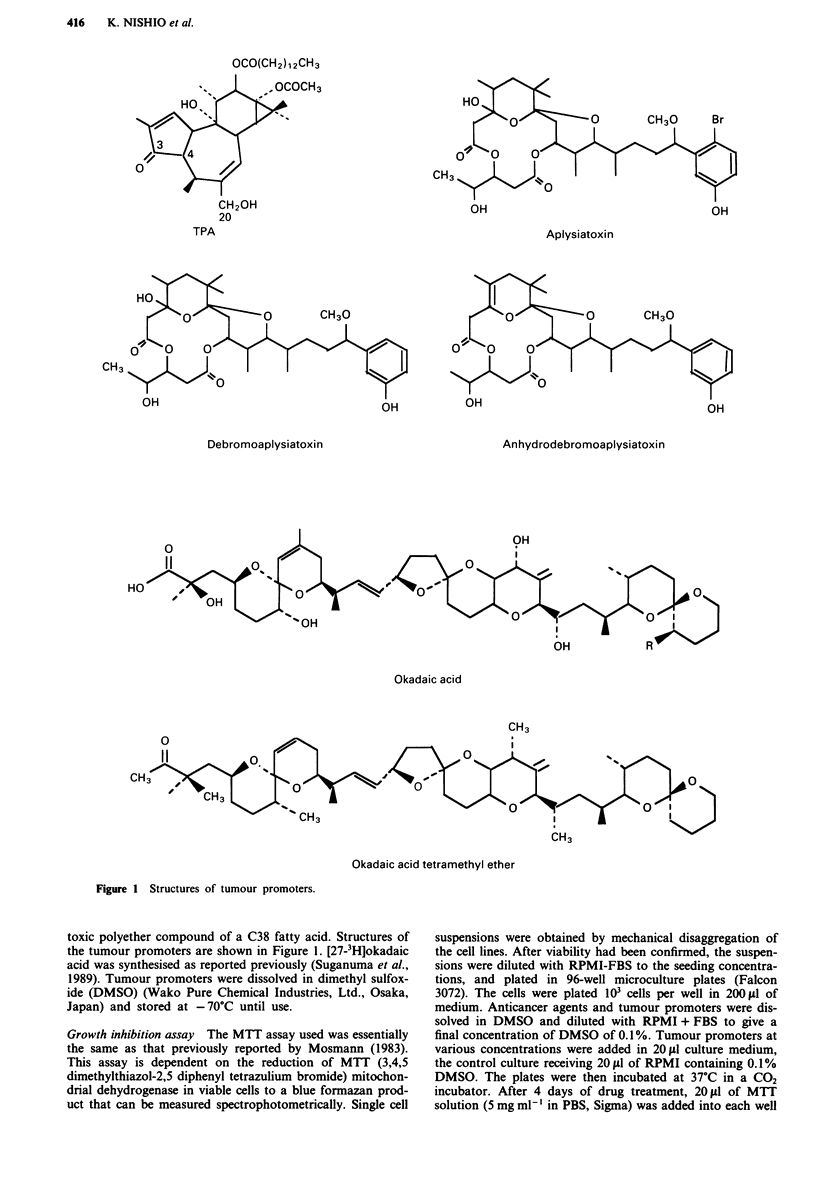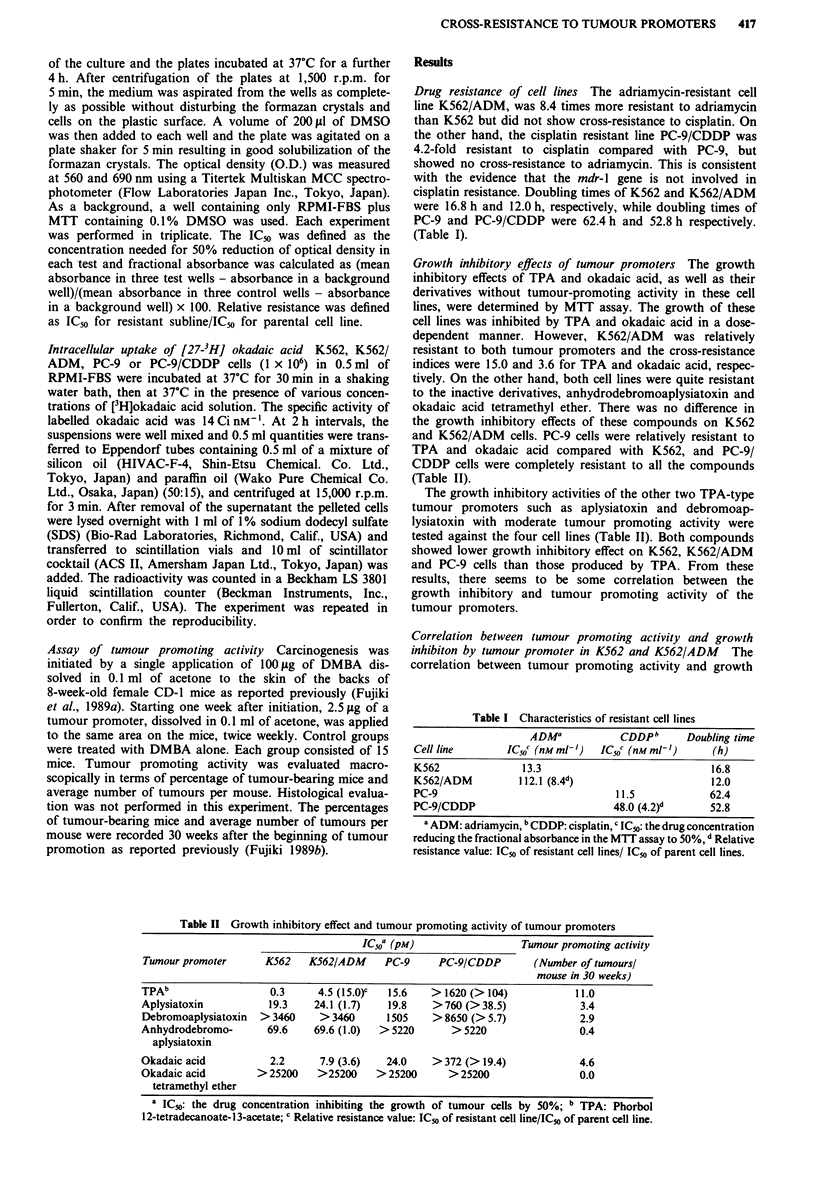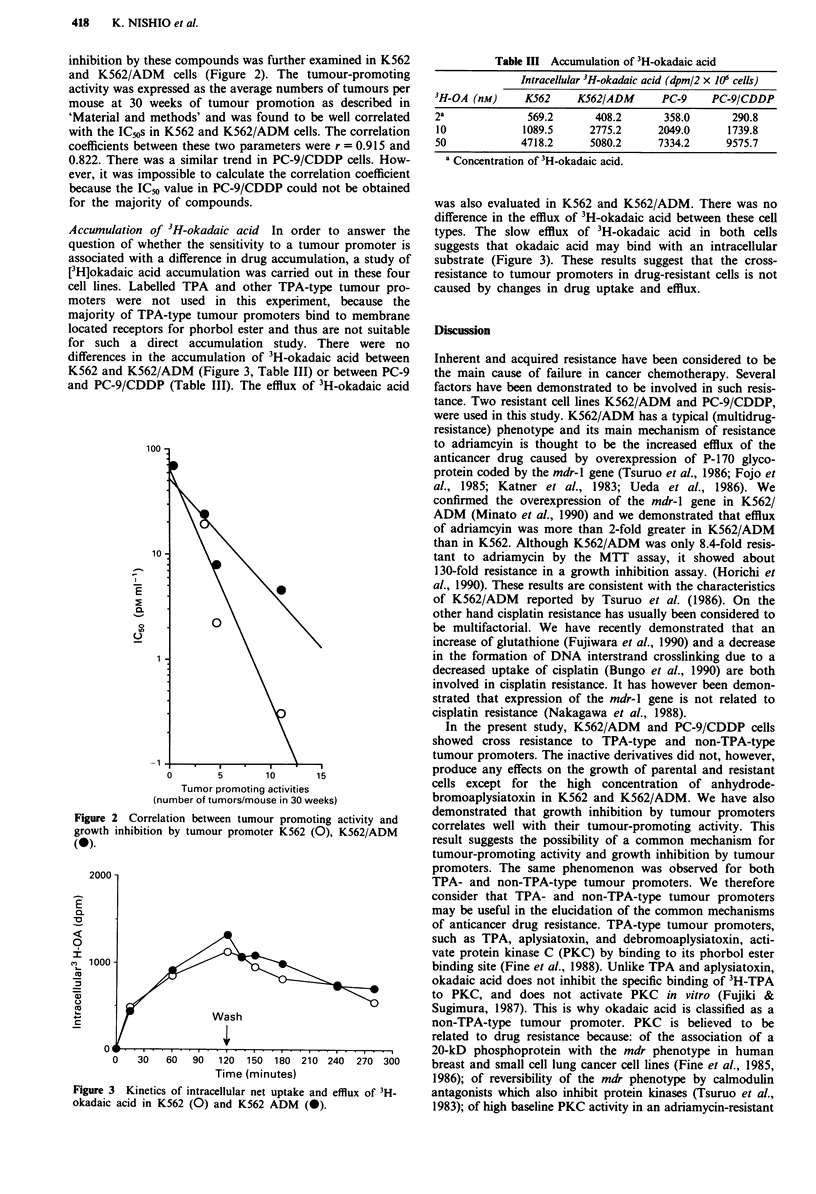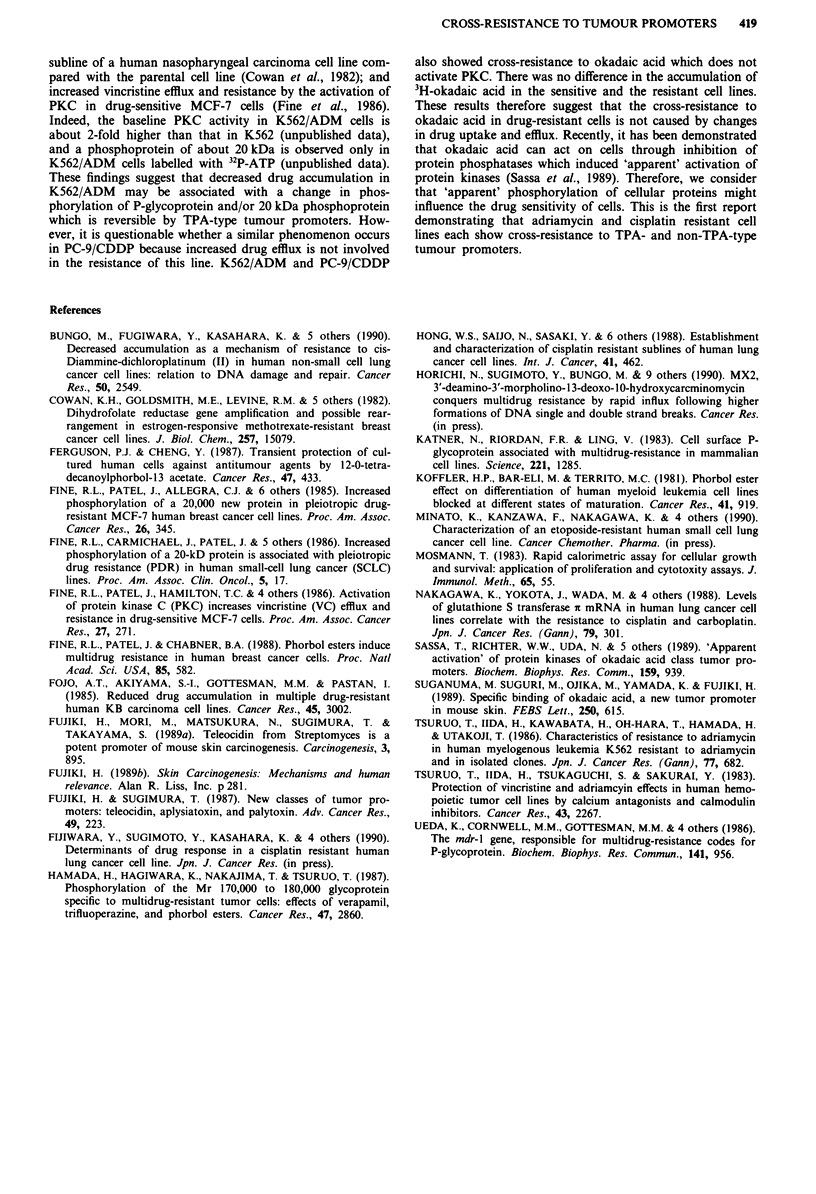# Cross-resistance to tumour promoters in human cancer cell lines resistant to adriamycin or cisplatin.

**DOI:** 10.1038/bjc.1990.309

**Published:** 1990-09

**Authors:** K. Nishio, Y. Sugimoto, K. Nakagawa, S. Niimi, Y. Fujiwara, M. Bungo, K. Kasahara, H. Fujiki, N. Saijo

**Affiliations:** Pharmacology Division, National Cancer Centre Research Institute, Tokyo, Japan.

## Abstract

The growth inhibitory effect of tumour promoters on human leukaemia and lung cancer cell lines was examined using the [3-(4,5 dimethylthiazol)-2, 5-diphenyl-tetrazolium bromide (MTT) assay. The four cell lines used were the K562 human leukaemia cell line, its adriamycin (ADM)-resistant subline (K562/ADM), which shows the mdr phenotype, PC-9 (a human lung adenocarcinoma cell line) and its cisplatin (CDDP)-resistant subline (PC-9/CDDP), which does not show the mdr phenotype. Phorbol 12-tetradecanoate-13-acetate (TPA) and the TPA-type tumour promoters, aplysiatoxin and debromoaplysiatoxin, inhibited the growth of the two parental cell lines, K562 and PC-9. The non-TPA-type tumour promoter, okadaic acid, also inhibited the growth of the two parental cell lines in a dose-dependent manner. TPA-type and okadaic acid inhibited the growth of K562/ADM more weakly than that of K562, and showed no growth inhibition in PC-9/CDDP. Anhydrodebromoaplysiatoxin, an inactive derivative of the TPA-type tumour promoter, could suppress the growth of K562 and K562/ADM only at high concentration (more than 50 pM) and it showed similar growth inhibitory effects on the two cell lines. Okadaic acid tetramethyl ether, the inactive form of the non-TPA-type tumour promoter did not inhibit the growth of any of the cell lines. The growth inhibitory effect of these compounds was well correlated with their tumour-promoting activity. A study of the accumulation of okadaic acid revealed that the amount of 3H-okadaic acid in K562/ADM and PC-9/CDDP was similar to that in their parental cells indicating that cross-resistance to this tumour promoter in the drug-resistant cell lines is not due to a difference in the amount of drug accumulated in sensitive and resistant cells. These results suggest the presence of another common mechanism for resistance to ADM and CDDP as well as to TPA- or non-TPA-type tumour promoters.


Br. J. Cancer (1990), 62, 415–419 © Macmillan Press Ltd., 1990

### Cross-resistance to tumour promoters in human cancer cell lines resistant to adriamycin or cisplatin

K. Nishio[1], Y. Sugimoto[1], K. Nakagawa[1], S. Niimi[1], Y. Fujiwara[1], M. Bungo[1], K. Kasahara[1], H. Fujiki[2] & N. Saijo[1]

[1]*Pharmacology Division &* [2]*Cancer Prevention Division, National Cancer Centre Research Insitute, Tsukiji 5-1-1, Chuo-ku, Tokyo 104, Japan.*

**Summary** The growth inhibitory effect of tumour promoters on human leukaemia and lung cancer cell lines was examined using the [3-(4,5 dimethylthiazol)-2, 5-diphenyl-tetrazolium bromide (MTT) assay. The four cell lines used were the K562 human leukaemia cell line, its adriamycin (ADM)-resistant subline (K562/ADM), which shows the *mdr* phenotype, PC-9 (a human lung adenocarcinoma cell line) and its cisplatin (CDDP)-resistant subline (PC-9/CDDP), which does not show the *mdr* phenotype. Phorbol 12-tetradecanoate-13-acetate (TPA) and the TPA-type tumour promoters, aplysiatoxin and debromoaplysiatoxin, inhibited the growth of the two parental cell lines, K562 and PC-9. The non-TPA-type tumour promoter, okadaic acid, also inhibited the growth of the two parental cell lines in a dose-dependent manner. TPA-type and okadaic acid inhibited the growth of K562/ADM more weakly than that of K562, and showed no growth inhibition in PC-9/CDDP. Anhydrodebromoaplysiatoxin, an inactive derivative of the TPA-type tumour promoter, could suppress the growth of K562 and K562/ADM only at high concentration (more than 50 pM) and it showed similar growth inhibitory effects on the two cell lines. Okadaic acid tetramethyl ether, the inactive form of the non-TPA-type tumour promoter did not inhibit the growth of any of the cell lines. The growth inhibitory effect of these compounds was well correlated with their tumour-promoting activity. A study of the accumulation of okadaic acid revealed that the amount of $^{3}H$-okadaic acid in K562/ADM and PC-9/CDDP was similar to that in their parental cells indicating that cross-resistance to this tumour promoter in the drug-resistant cell lines is not due to a difference in the amount of drug accumulated in sensitive and resistant cells. These results suggest the presence of another common mechanism for resistance to ADM and CDDP as well as to TPA- or non-TPA-type tumour promoters.

Phorbol esters such as TPA have several biological activities. TPA activates protein kinase C (PKC) which has been considered to be a key enzyme involved in promotion, cell differentiation and proliferation. On the other hand, there are several reports that activation of protein kinase C by phorbol ester modulates resistance to anticancer drugs such as vincristine and etoposide in breast cancer or small cell carcinoma cell lines (Ferguson & Cheng, 1987). Modulation of drug resistance by TPA is possibly due to phosphorylation of P-glycoprotein, although the modulatory effects are transient (Hamada *et al.*, 1987). To investigate this, we examined the sensitizing effects of TPA to anticancer drugs using the K562/ADM cell line which possesses the *mdr* phenotype and its parental cell line K562. Modulatory effects of TPA were not however observed in this assay system (unpublished data). However, in the course of these studies, we observed that TPA showed a growth inhibitory effect on K562 and K562/ADM cells but did not induce differentiation in these cells. Because of this result, we considered that these cells may be useful to study the growth inhibitory effects of tumour promoters in the absence of cell differentiation. We also found TPA to have different inhibitory effects on the growth of parental and drug-resistant cell lines. K562/ADM and PC-9/CDDP revealed cross-resistance to TPA. These data suggested that tumour promoters may be useful compounds in the elucidation of new mechanisms of drug resistance.

On the other hand, recent reports suggest that some compounds such as bryostatin II, which have activity resembling that of phorbol ester, show cytotoxic effects towards some tumour cells. The mechanisms of antitumour activity of these compounds are considered to be activation of a specific isozyme of PKC, or down regulation of PKC. We considered that the elucidation of the relationship between the growth inhibitory effects and the tumour promoting activity of tumour promoters would be useful not only for the development of new anticancer drugs, but also for the elucidation of unknown mechanisms of resistance to drugs. We have, therefore, examined the growth inhibitory effect of several tumour promoters on drug sensitive and resistant cell lines.

Correspondence: N. Saijo.
Received 9 February 1990; and in revised form 5 April 1990.

### Materials and methods

*Cell lines* The human leukaemia cell line, K562, and the human lung cancer cell line, PC-9 together with their drug-resistant sublines were used for the study. K562 was established from a patient with chronic myelocytic leukaemia. Its adriamycin-resistant subline, K562/ADM, was kindly donated by Professor T. Tsuruo, Tokyo University. PC-9, derived from a previously untreated patient with adenocarcinoma of the lung, was provided by Professor Y. Hayata, Tokyo Medical College. Its cisplatin-resistant subline, PC-9/CDDP was established by continuous exposure to a stepwise-increasing concentration of cisplatin (to 0.1 $\mu g\, ml^{-1}$) and selection by limiting dilution technique. This cell line showed 4.2-fold resistance to cisplatin compared to the respective parental cell line, PC-9, based on the 50% inhibitory concentrations ($IC_{50}$) in a tetrazolium dye (MTT) assay with continuous drug exposure (Hong *et al.*, 1988). All cell lines grew in RPMI 1640 medium (Gibco Laboratories, Tokyo Japan) supplemented with 10% fetal bovine serum (Gibco), penicillin (100 U $ml^{-1}$) and streptomycin (100 $\mu g\, ml^{-1}$) (RPMI-FBS) in a humidified 5% $CO_2$ atmosphere at 37°C. All cell lines grew mainly as floating aggregates, although PC-9 was also partially attached to the surface of the culture flask. The lines were subcultured once or twice per week.

*Tumour promoters* TPA and the TPA-type tumour promoters, aplysiatoxin and debromoaplysiatoxin, and a non-TPA-type tumour promoter, okadaic acid, were used in these experiments. Anhydrodebromoaplysiatoxin and okadaic acid tetramethyl ether were used as inactive, control compounds. TPA was purchased from Sigma Chemical Co. St. Louis MO. Other tumour promoters and their derivatives, aplysiatoxin and debromoaplysiatoxin have an acetogenic, phenolic bislactone structure (Figure 1). Okadaic acid is a

**Figure 1** Structures of tumour promoters.

toxic polyether compound of a C38 fatty acid. Structures of the tumour promoters are shown in Figure 1. [27-$^3$H]okadaic acid was synthesised as reported previously (Suganuma *et al.*, 1989). Tumour promoters were dissolved in dimethyl sulfoxide (DMSO) (Wako Pure Chemical Industries, Ltd., Osaka, Japan) and stored at −70°C until use.

*Growth inhibition assay* The MTT assay used was essentially the same as that previously reported by Mosmann (1983). This assay is dependent on the reduction of MTT (3,4,5 dimethylthiazol-2,5 diphenyl tetrazulium bromide) mitochondrial dehydrogenase in viable cells to a blue formazan product that can be measured spectrophotometrically. Single cell suspensions were obtained by mechanical disaggregation of the cell lines. After viability had been confirmed, the suspensions were diluted with RPMI-FBS to the seeding concentrations, and plated in 96-well microculture plates (Falcon 3072). The cells were plated $10^3$ cells per well in 200 μl of medium. Anticancer agents and tumour promoters were dissolved in DMSO and diluted with RPMI + FBS to give a final concentration of DMSO of 0.1%. Tumour promoters at various concentrations were added in 20 μl culture medium, the control culture receiving 20 μl of RPMI containing 0.1% DMSO. The plates were then incubated at 37°C in a $CO_2$ incubator. After 4 days of drug treatment, 20 μl of MTT solution (5 mg $ml^{-1}$ in PBS, Sigma) was added into each well of the culture and the plates incubated at 37°C for a further 4 h. After centrifugation of the plates at 1,500 r.p.m. for 5 min, the medium was aspirated from the wells as completely as possible without disturbing the formazan crystals and cells on the plastic surface. A volume of 200 μl of DMSO was then added to each well and the plate was agitated on a plate shaker for 5 min resulting in good solubilization of the formazan crystals. The optical density (O.D.) was measured at 560 and 690 nm using a Titertek Multiskan MCC spectrophotometer (Flow Laboratories Japan Inc., Tokyo, Japan). As a background, a well containing only RPMI-FBS plus MTT containing 0.1% DMSO was used. Each experiment was performed in triplicate. The $IC_{50}$ was defined as the concentration needed for 50% reduction of optical density in each test and fractional absorbance was calculated as (mean absorbance in three test wells - absorbance in a background well)/(mean absorbance in three control wells - absorbance in a background well) × 100. Relative resistance was defined as $IC_{50}$ for resistant subline/$IC_{50}$ for parental cell line.

*Intracellular uptake of [27-³H] okadaic acid* K562, K562/ADM, PC-9 or PC-9/CDDP cells ($1 \times 10^6$) in 0.5 ml of RPMI-FBS were incubated at 37°C for 30 min in a shaking water bath, then at 37°C in the presence of various concentrations of [³H]okadaic acid solution. The specific activity of labelled okadaic acid was 14 Ci $nM^{-1}$. At 2 h intervals, the suspensions were well mixed and 0.5 ml quantities were transferred to Eppendorf tubes containing 0.5 ml of a mixture of silicon oil (HIVAC-F-4, Shin-Etsu Chemical. Co. Ltd., Tokyo, Japan) and paraffin oil (Wako Pure Chemical Co. Ltd., Osaka, Japan) (50:15), and centrifuged at 15,000 r.p.m. for 3 min. After removal of the supernatant the pelleted cells were lysed overnight with 1 ml of 1% sodium dodecyl sulfate (SDS) (Bio-Rad Laboratories, Richmond, Calif., USA) and transferred to scintillation vials and 10 ml of scintillator cocktail (ACS II, Amersham Japan Ltd., Tokyo, Japan) was added. The radioactivity was counted in a Beckham LS 3801 liquid scintillation counter (Beckman Instruments, Inc., Fullerton, Calif., USA). The experiment was repeated in order to confirm the reproducibility.

*Assay of tumour promoting activity* Carcinogenesis was initiated by a single application of 100 μg of DMBA dissolved in 0.1 ml of acetone to the skin of the backs of 8-week-old female CD-1 mice as reported previously (Fujiki *et al.*, 1989*a*). Starting one week after initiation, 2.5 μg of a tumour promoter, dissolved in 0.1 ml of acetone, was applied to the same area on the mice, twice weekly. Control groups were treated with DMBA alone. Each group consisted of 15 mice. Tumour promoting activity was evaluated macroscopically in terms of percentage of tumour-bearing mice and average number of tumours per mouse. Histological evaluation was not performed in this experiment. The percentages of tumour-bearing mice and average number of tumours per mouse were recorded 30 weeks after the beginning of tumour promotion as reported previously (Fujiki 1989*b*).

### Results

*Drug resistance of cell lines* The adriamycin-resistant cell line K562/ADM, was 8.4 times more resistant to adriamycin than K562 but did not show cross-resistance to cisplatin. On the other hand, the cisplatin resistant line PC-9/CDDP was 4.2-fold resistant to cisplatin compared with PC-9, but showed no cross-resistance to adriamycin. This is consistent with the evidence that the *mdr*-1 gene is not involved in cisplatin resistance. Doubling times of K562 and K562/ADM were 16.8 h and 12.0 h, respectively, while doubling times of PC-9 and PC-9/CDDP were 62.4 h and 52.8 h respectively. (Table I).

*Growth inhibitory effects of tumour promoters* The growth inhibitory effects of TPA and okadaic acid, as well as their derivatives without tumour-promoting activity in these cell lines, were determined by MTT assay. The growth of these cell lines was inhibited by TPA and okadaic acid in a dose-dependent manner. However, K562/ADM was relatively resistant to both tumour promoters and the cross-resistance indices were 15.0 and 3.6 for TPA and okadaic acid, respectively. On the other hand, both cell lines were quite resistant to the inactive derivatives, anhydrodebromoaplysiatoxin and okadaic acid tetramethyl ether. There was no difference in the growth inhibitory effects of these compounds on K562 and K562/ADM cells. PC-9 cells were relatively resistant to TPA and okadaic acid compared with K562, and PC-9/CDDP cells were completely resistant to all the compounds (Table II).

The growth inhibitory activities of the other two TPA-type tumour promoters such as aplysiatoxin and debromoaplysiatoxin with moderate tumour promoting activity were tested against the four cell lines (Table II). Both compounds showed lower growth inhibitory effect on K562, K562/ADM and PC-9 cells than those produced by TPA. From these results, there seems to be some correlation between the growth inhibitory and tumour promoting activity of the tumour promoters.

*Correlation between tumour promoting activity and growth inhibiton by tumour promoter in K562 and K562/ADM* The correlation between tumour promoting activity and growth

**Table I** Characteristics of resistant cell lines

| Cell line | ADM[a] $IC_{50}$[c] ($nM\ ml^{-1}$) | CDDP[b] $IC_{50}$[c] ($nM\ ml^{-1}$) | Doubling time (h) |
|---|---|---|---|
| K562 | 13.3 | | 16.8 |
| K562/ADM | 112.1 (8.4[d]) | | 12.0 |
| PC-9 | | 11.5 | 62.4 |
| PC-9/CDDP | | 48.0 (4.2)[d] | 52.8 |

[a] ADM: adriamycin, [b] CDDP: cisplatin, [c] $IC_{50}$: the drug concentration reducing the fractional absorbance in the MTT assay to 50%, [d] Relative resistance value: $IC_{50}$ of resistant cell lines/ $IC_{50}$ of parent cell lines.

**Table II** Growth inhibitory effect and tumour promoting activity of tumour promoters

| Tumour promoter | $IC_{50}$[a] (pM) K562 | K562/ADM | PC-9 | PC-9/CDDP | Tumour promoting activity (Number of tumours/ mouse in 30 weeks) |
|---|---|---|---|---|---|
| TPA[b] | 0.3 | 4.5 (15.0)[c] | 15.6 | >1620 (>104) | 11.0 |
| Aplysiatoxin | 19.3 | 24.1 (1.7) | 19.8 | >760 (>38.5) | 3.4 |
| Debromoaplysiatoxin | >3460 | >3460 | 1505 | >8650 (>5.7) | 2.9 |
| Anhydrodebromo-aplysiatoxin | 69.6 | 69.6 (1.0) | >5220 | >5220 | 0.4 |
| Okadaic acid | 2.2 | 7.9 (3.6) | 24.0 | >372 (>19.4) | 4.6 |
| Okadaic acid tetramethyl ether | >25200 | >25200 | >25200 | >25200 | 0.0 |

[a] $IC_{50}$: the drug concentration inhibiting the growth of tumour cells by 50%; [b] TPA: Phorbol 12-tetradecanoate-13-acetate; [c] Relative resistance value: $IC_{50}$ of resistant cell line/$IC_{50}$ of parent cell line.

inhibition by these compounds was further examined in K562 and K562/ADM cells (Figure 2). The tumour-promoting activity was expressed as the average numbers of tumours per mouse at 30 weeks of tumour promotion as described in 'Material and methods' and was found to be well correlated with the $IC_{50}$s in K562 and K562/ADM cells. The correlation coefficients between these two parameters were $r = 0.915$ and 0.822. There was a similar trend in PC-9/CDDP cells. However, it was impossible to calculate the correlation coefficient because the $IC_{50}$ value in PC-9/CDDP could not be obtained for the majority of compounds.

*Accumulation of $^3$H-okadaic acid* In order to answer the question of whether the sensitivity to a tumour promoter is associated with a difference in drug accumulation, a study of [$^3$H]okadaic acid accumulation was carried out in these four cell lines. Labelled TPA and other TPA-type tumour promoters were not used in this experiment, because the majority of TPA-type tumour promoters bind to membrane located receptors for phorbol ester and thus are not suitable for such a direct accumulation study. There were no differences in the accumulation of $^3$H-okadaic acid between K562 and K562/ADM (Figure 3, Table III) or between PC-9 and PC-9/CDDP (Table III). The efflux of $^3$H-okadaic acid was also evaluated in K562 and K562/ADM. There was no difference in the efflux of $^3$H-okadaic acid between these cell types. The slow efflux of $^3$H-okadaic acid in both cells suggests that okadaic acid may bind with an intracellular substrate (Figure 3). These results suggest that the cross-resistance to tumour promoters in drug-resistant cells is not caused by changes in drug uptake and efflux.

**Figure 2** Correlation between tumour promoting activity and growth inhibition by tumour promoter K562 (○), K562/ADM (●).

**Figure 3** Kinetics of intracellular net uptake and efflux of $^3$H-okadaic acid in K562 (○) and K562 ADM (●).

**Table III** Accumulation of $^3$H-okadaic acid

| | *Intracellular $^3$H-okadaic acid (dpm/2 × $10^6$ cells)* | | | |
|---|---|---|---|---|
| *$^3$H-OA (nM)* | *K562* | *K562/ADM* | *PC-9* | *PC-9/CDDP* |
| 2[a] | 569.2 | 408.2 | 358.0 | 290.8 |
| 10 | 1089.5 | 2775.2 | 2049.0 | 1739.8 |
| 50 | 4718.2 | 5080.2 | 7334.2 | 9575.7 |

[a] Concentration of $^3$H-okadaic acid.

### Discussion

Inherent and acquired resistance have been considered to be the main cause of failure in cancer chemotherapy. Several factors have been demonstrated to be involved in such resistance. Two resistant cell lines K562/ADM and PC-9/CDDP, were used in this study. K562/ADM has a typical (multidrug-resistance) phenotype and its main mechanism of resistance to adriamcyin is thought to be the increased efflux of the anticancer drug caused by overexpression of P-170 glycoprotein coded by the *mdr*-1 gene (Tsuruo *et al.*, 1986; Fojo *et al.*, 1985; Katner *et al.*, 1983; Ueda *et al.*, 1986). We confirmed the overexpression of the *mdr*-1 gene in K562/ADM (Minato *et al.*, 1990) and we demonstrated that efflux of adriamcyin was more than 2-fold greater in K562/ADM than in K562. Although K562/ADM was only 8.4-fold resistant to adriamycin by the MTT assay, it showed about 130-fold resistance in a growth inhibition assay. (Horichi *et al.*, 1990). These results are consistent with the characteristics of K562/ADM reported by Tsuruo *et al.* (1986). On the other hand cisplatin resistance has usually been considered to be multifactorial. We have recently demonstrated that an increase of glutathione (Fujiwara *et al.*, 1990) and a decrease in the formation of DNA interstrand crosslinking due to a decreased uptake of cisplatin (Bungo *et al.*, 1990) are both involved in cisplatin resistance. It has however been demonstrated that expression of the *mdr*-1 gene is not related to cisplatin resistance (Nakagawa *et al.*, 1988).

In the present study, K562/ADM and PC-9/CDDP cells showed cross resistance to TPA-type and non-TPA-type tumour promoters. The inactive derivatives did not, however, produce any effects on the growth of parental and resistant cells except for the high concentration of anhydrodebromoaplysiatoxin in K562 and K562/ADM. We have also demonstrated that growth inhibition by tumour promoters correlates well with their tumour-promoting activity. This result suggests the possibility of a common mechanism for tumour-promoting activity and growth inhibition by tumour promoters. The same phenomenon was observed for both TPA- and non-TPA-type tumour promoters. We therefore consider that TPA- and non-TPA-type tumour promoters may be useful in the elucidation of the common mechanisms of anticancer drug resistance. TPA-type tumour promoters, such as TPA, aplysiatoxin, and debromoaplysiatoxin, activate protein kinase C (PKC) by binding to its phorbol ester binding site (Fine *et al.*, 1988). Unlike TPA and aplysiatoxin, okadaic acid does not inhibit the specific binding of $^3$H-TPA to PKC, and does not activate PKC *in vitro* (Fujiki & Sugimura, 1987). This is why okadaic acid is classified as a non-TPA-type tumour promoter. PKC is believed to be related to drug resistance because: of the association of a 20-kD phosphoprotein with the *mdr* phenotype in human breast and small cell lung cancer cell lines (Fine *et al.*, 1985, 1986); of reversibility of the *mdr* phenotype by calmodulin antagonists which also inhibit protein kinases (Tsuruo *et al.*, 1983); of high baseline PKC activity in an adriamycin-resistant subline of a human nasopharyngeal carcinoma cell line compared with the parental cell line (Cowan *et al.*, 1982); and increased vincristine efflux and resistance by the activation of PKC in drug-sensitive MCF-7 cells (Fine *et al.*, 1986). Indeed, the baseline PKC activity in K562/ADM cells is about 2-fold higher than that in K562 (unpublished data), and a phosphoprotein of about 20 kDa is observed only in K562/ADM cells labelled with $^{32}$P-ATP (unpublished data). These findings suggest that decreased drug accumulation in K562/ADM may be associated with a change in phosphorylation of P-glycoprotein and/or 20 kDa phosphoprotein which is reversible by TPA-type tumour promoters. However, it is questionable whether a similar phenomenon occurs in PC-9/CDDP because increased drug efflux is not involved in the resistance of this line. K562/ADM and PC-9/CDDP also showed cross-resistance to okadaic acid which does not activate PKC. There was no difference in the accumulation of $^{3}$H-okadaic acid in the sensitive and the resistant cell lines. These results therefore suggest that the cross-resistance to okadaic acid in drug-resistant cells is not caused by changes in drug uptake and efflux. Recently, it has been demonstrated that okadaic acid can act on cells through inhibition of protein phosphatases which induced 'apparent' activation of protein kinases (Sassa *et al.*, 1989). Therefore, we consider that 'apparent' phosphorylation of cellular proteins might influence the drug sensitivity of cells. This is the first report demonstrating that adriamycin and cisplatin resistant cell lines each show cross-resistance to TPA- and non-TPA-type tumour promoters.

### References

BUNGO, M., FUGIWARA, Y., KASAHARA, K. & 5 others (1990). Decreased accumulation as a mechanism of resistance to cis-Diammine-dichloroplatinum (II) in human non-small cell lung cancer cell lines: relation to DNA damage and repair. *Cancer Res.*, **50**, 2549.

COWAN, K.H., GOLDSMITH, M.E., LEVINE, R.M. & 5 others (1982). Dihydrofolate reductase gene amplification and possible rearrangement in estrogen-responsive methotrexate-resistant breast cancer cell lines. *J. Biol. Chem.*, **257**, 15079.

FERGUSON, P.J. & CHENG, Y. (1987). Transient protection of cultured human cells against antitumour agents by 12-0-tetradecanoylphorbol-13 acetate. *Cancer Res.*, **47**, 433.

FINE, R.L., PATEL, J., ALLEGRA, C.J. & 6 others (1985). Increased phosphorylation of a 20,000 new protein in pleiotropic drug-resistant MCF-7 human breast cancer cell lines. *Proc. Am. Assoc. Cancer Res.*, **26**, 345.

FINE, R.L., CARMICHAEL, J., PATEL, J. & 5 others (1986). Increased phosphorylation of a 20-kD protein is associated with pleiotropic drug resistance (PDR) in human small-cell lung cancer (SCLC) lines. *Proc. Am. Assoc. Clin. Oncol.*, **5**, 17.

FINE, R.L., PATEL, J., HAMILTON, T.C. & 4 others (1986). Activation of protein kinase C (PKC) increases vincristine (VC) efflux and resistance in drug-sensitive MCF-7 cells. *Proc. Am. Assoc. Cancer Res.*, **27**, 271.

FINE, R.L., PATEL, J. & CHABNER, B.A. (1988). Phorbol esters induce multidrug resistance in human breast cancer cells. *Proc. Natl Acad. Sci. USA*, **85**, 582.

FOJO, A.T., AKIYAMA, S.-I., GOTTESMAN, M.M. & PASTAN, I. (1985). Reduced drug accumulation in multiple drug-resistant human KB carcinoma cell lines. *Cancer Res.*, **45**, 3002.

FUJIKI, H., MORI, M., MATSUKURA, N., SUGIMURA, T. & TAKAYAMA, S. (1989*a*). Teleocidin from Streptomyces is a potent promoter of mouse skin carcinogenesis. *Carcinogenesis*, **3**, 895.

FUJIKI, H. (1989*b*). *Skin Carcinogenesis: Mechanisms and human relevance*. Alan R. Liss, Inc. p 281.

FUJIKI, H. & SUGIMURA, T. (1987). New classes of tumor promoters: teleocidin, aplysiatoxin, and palytoxin. *Adv. Cancer Res.*, **49**, 223.

FIJIWARA, Y., SUGIMOTO, Y., KASAHARA, K. & 4 others (1990). Determinants of drug response in a cisplatin resistant human lung cancer cell line. *Jpn. J. Cancer Res.* (in press).

HAMADA, H., HAGIWARA, K., NAKAJIMA, T. & TSURUO, T. (1987). Phosphorylation of the Mr 170,000 to 180,000 glycoprotein specific to multidrug-resistant tumor cells: effects of verapamil, trifluoperazine, and phorbol esters. *Cancer Res.*, **47**, 2860.

HONG, W.S., SAIJO, N., SASAKI, Y. & 6 others (1988). Establishment and characterization of cisplatin resistant sublines of human lung cancer cell lines. *Int. J. Cancer*, **41**, 462.

HORICHI, N., SUGIMOTO, Y., BUNGO, M. & 9 others (1990). MX2, 3′-deamino-3′-morpholino-13-deoxo-10-hydroxycarcminomycin conquers multidrug resistance by rapid influx following higher formations of DNA single and double strand breaks. *Cancer Res.* (in press).

KATNER, N., RIORDAN, F.R. & LING, V. (1983). Cell surface P-glycoprotein associated with multidrug-resistance in mammalian cell lines. *Science*, **221**, 1285.

KOFFLER, H.P., BAR-ELI, M. & TERRITO, M.C. (1981). Phorbol ester effect on differentiation of human myeloid leukemia cell lines blocked at different states of maturation. *Cancer Res.*, **41**, 919.

MINATO, K., KANZAWA, F., NAKAGAWA, K. & 4 others (1990). Characterization of an etoposide-resistant human small cell lung cancer cell line. *Cancer Chemother. Pharma.* (in press).

MOSMANN, T. (1983). Rapid calorimetric assay for cellular growth and survival: application of proliferation and cytotoxity assays. *J. Immunol. Meth.*, **65**, 55.

NAKAGAWA, K., YOKOTA, J., WADA, M. & 4 others (1988). Levels of glutathione S transferase π mRNA in human lung cancer cell lines correlate with the resistance to cisplatin and carboplatin. *Jpn. J. Cancer Res. (Gann)*, **79**, 301.

SASSA, T., RICHTER, W.W., UDA, N. & 5 others (1989). 'Apparent activation' of protein kinases of okadaic acid class tumor promoters. *Biochem. Biophys. Res. Comm.*, **159**, 939.

SUGANUMA, M. SUGURI, M., OJIKA, M., YAMADA, K. & FUJIKI, H. (1989). Specific binding of okadaic acid, a new tumor promoter in mouse skin. *FEBS Lett.*, **250**, 615.

TSURUO, T., IIDA, H., KAWABATA, H., OH-HARA, T., HAMADA, H. & UTAKOJI, T. (1986). Characteristics of resistance to adriamycin in human myelogenous leukemia K562 resistant to adriamycin and in isolated clones. *Jpn. J. Cancer Res. (Gann)*, **77**, 682.

TSURUO, T., IIDA, H., TSUKAGUCHI, S. & SAKURAI, Y. (1983). Protection of vincristine and adriamcyin effects in human hemopoietic tumor cell lines by calcium antagonists and calmodulin inhibitors. *Cancer Res.*, **43**, 2267.

UEDA, K., CORNWELL, M.M., GOTTESMAN, M.M. & 4 others (1986). The *mdr*-1 gene, responsible for multidrug-resistance codes for P-glycoprotein. *Biochem. Biophys. Res. Commun.*, **141**, 956.